# Interpreting clinical trial data in multiple myeloma: translating findings to the real-world setting

**DOI:** 10.1038/s41408-018-0141-0

**Published:** 2018-11-09

**Authors:** Paul G. Richardson, Jesus F. San Miguel, Philippe Moreau, Roman Hajek, Meletios A. Dimopoulos, Jacob P. Laubach, Antonio Palumbo, Katarina Luptakova, Dorothy Romanus, Tomas Skacel, Shaji K. Kumar, Kenneth C. Anderson

**Affiliations:** 1 Medical Oncology, Dana-Farber Cancer Institute, Boston, MA USA; 2 Centro Investigación Medica Aplicada (CIMA), IDISNA; CIBERONC, Clinica Universidad de Navarra, Pamplona, Spain; 3 Department of Hematology, University Hospital Hôtel Dieu, Nantes, France; 4 Department of Hematooncology, University Hospital Ostrava, Ostrava, Czech Republic; 5 Department of Clinical Therapeutics, School of Medicine, National and Kapodistrian University of Athens, Athens, Greece; 6 Millennium Pharmaceuticals, Inc., a wholly owned subsidiary of Takeda Pharmaceutical Company Limited, Cambridge, MA USA; 7 Division of Hematology, Mayo Clinic, Rochester, MN USA

## Abstract

Substantial improvements in survival have been seen in multiple myeloma (MM) over recent years, associated with the introduction and widespread use of multiple novel agents and regimens, as well as the emerging treatment paradigm of continuous or long-term therapy. However, these therapies and approaches may have limitations in the community setting, associated with toxicity burden, patient burden, and other factors including cost. Consequently, despite improvements in efficacy in the rigorously controlled clinical trials setting, the same results are not always achieved in real-world practice. Furthermore, the large number of different treatment options and regimens under investigation in various MM settings precludes the feasibility of obtaining head-to-head clinical trial data, and there is a temptation to use cross-trial comparisons to evaluate data across regimens. However, multiple aspects, including patient-related, disease-related, and treatment-related factors, can influence clinical trial outcomes and lead to differences between studies that may confound direct comparisons between data. In this review, we explore the various factors requiring attention when evaluating clinical trial data across available agents/regimens, as well as other considerations that may impact the translation of these findings into everyday MM management. We also investigate discrepancies between clinical trial efficacy and real-world effectiveness through a literature review of non-clinical trial data in relapsed/refractory MM on novel agent−based regimens and evaluate these data in the context of phase 3 trial results for recently approved and commonly used regimens. We thereby demonstrate the complexity of interpreting data across clinical studies in MM, as well as between clinical studies and routine-care analyses, with the aim to help clinicians consider all the necessary issues when tailoring individual patients’ treatment approaches.

## Introduction

Substantial improvements in overall survival (OS) have been seen in multiple myeloma (MM) over recent years^1^, with data from the United States National Cancer Institute Surveillance, Epidemiology, and End Results Program demonstrating a 5-year OS rate of almost 50%^2^ and an increase in the 10-year OS rate among patients diagnosed in 1990 and in 2004 from 10% to 26.8%^3^. These improvements are associated with the introduction and widespread use of multiple novel agents and regimens^4^, as well as with the emerging treatment paradigm of continuous therapy or long-term maintenance therapy^5–8^, an approach that has been shown in clinical trials to offer prolonged survival versus fixed-duration or shorter-term therapeutic approaches^6–9^.

However, there is the potential for discrepancies between clinical trial efficacy, for which an intervention is studied under ideal circumstances, and real-world effectiveness, which reflects the true benefit of an intervention to patients in routine practice. This is associated with a number of factors and confounds translation of clinical trial data into the real world. For example, continuous or long-term therapy may have limitations in routine clinical practice, associated with toxicity burden, patient burden, and other factors such as cost^5,10,11^. Strict patient selection criteria in clinical trials is another important aspect and is the subject of a recent American Society of Clinical Oncology initiative to broaden clinical trial eligibility criteria to be more representative of patient populations^12^. Determination of the appropriate regimen for MM patients in clinical practice requires individualized assessment of various patient-related, disease-related, and treatment-related characteristics. The gap between efficacy and effectiveness is also associated with toxicity and comorbidity burden, patient and physician motivation, different distributions of academic versus community centers at which patients receive their treatment, strict protocol-enforced surveillance, treatment access issues, and other determinants contributing to premature discontinuation of treatment regimens outside of clinical studies.

A related issue is that relative efficacy in the clinical trial setting represents a key consideration for regimen selection. There is a temptation to use cross-trial comparisons to compare data across regimens; however, many factors can influence clinical trial outcomes, and there may be differences between studies that may confound indirect comparisons. Indirect comparisons of relative efficacy across homogeneous study populations, such as the network meta-analysis approach recently utilized to compare PFS across multiple treatment options for RRMM^13^, using hazard ratios (HRs) may prove more valid in this context. Nonetheless, while rigorously conducted network meta-analyses are useful to inform treatment decisions, known limitations, including publication bias, study heterogeneity, and differences in study populations and median follow-up across clinical trials, may still lead to biased estimates of the comparative treatment effects^14^.

This review explores the various factors that physicians need to consider when evaluating clinical trial data across agents and regimens, focusing on studies in relapsed/refractory MM (RRMM), and addresses considerations regarding how these findings will translate into everyday MM management.

## Recent approvals in RRMM

Regulatory approvals based upon the results from randomized, phase 3 clinical trials remain the gold standard. Table 1 summarizes phase 3 clinical trial results for recently approved regimens for RRMM. These include monoclonal antibodies (daratumumab^15,16^ and elotuzumab^17^), proteasome inhibitors (intravenously administered [IV] carfilzomib^18,19^ and orally administered ixazomib^20^), and the histone deacetylase inhibitor panobinostat^21,22^. Real-world data in this setting are emerging for carfilzomib and ixazomib^23–26^, but such data are unfortunately limited for monoclonal antibodies^27,28^ and panobinostat to date^29,30^.

**Table 1:** Novel agents with new/updated approvals for the treatment of RRMM within the past 3 years, plus key efficacy findings from phase 3 studies leading to regulatory approvals

Agent	Indication	Study	Regimen	*N*	ORR, %	≥ VGPR, %	PFS, mos	OS, mos
RRMM								
Carfilzomib (IV proteasome inhibitor)	Approved in combination with dexamethasone (Kd)^18^ or lenalidomide-dexamethasone (KRd)^19^ for the treatment of MM patients following 1–3 prior therapies	ASPIRE^19,96^	KRd	396	87	70	26.3	48.3
			Rd	396	67	40	17.6	40.4
		ENDEAVOR^18,97^	Kd	464	77	54	18.7	47.6
			Vd	465	63	29	9.4	40.0
Ixazomib (oral proteasome inhibitor)	Approved in combination with lenalidomide-dexamethasone (IRd)^20^ for the treatment of MM patients after at least one prior therapy	TOURMALINE-MM1^20^	IRd	360	78	48	20.6	NR
			Placebo-Rd	362	72	39	14.7	NR
Elotuzumab (Anti-SLAMF7 monoclonal antibody)	Approved in combination with lenalidomide-dexamethasone (ERd)^17^ for the treatment of MM patients following 1–3 prior therapies	ELOQUENT-2^17,98^	ERd	321	79	33	19.4	48.0
			Rd	325	66	28	14.9	40.0
Daratumumab (Anti-CD38 monoclonal antibody)	Approved in combination with lenalidomide-dexamethasone (Dara-Rd)^15^ or bortezomib-dexamethasone (Dara-Vd)^16^ for the treatment of patients who have received at least one prior line of therapy	POLLUX^15,99^	Dara-Rd	286	93	79	NE	NR
			Rd	283	76	48	17.5	NR
		CASTOR^16,100^	Dara-Vd	251	83	59	NE	NE
			Vd	247	63	29	7.2	NE
Panobinostat (Histone deacetlyase inhibitor)	Approved for the treatment of patients with relapsed and/or refractory MM who have received at least two prior regimens, including bortezomib and an immunomodulatory agent	PANORAMA1^21,22^	Pan-Vd	387	61	NR	12.0	40.3
			Placebo-Vd	381	55	NR	8.1	35.8

*ASCT* autologous stem cell transplantation, *CR* complete response, *IFM* Intergroupe Francophone du Myelome, *mos* months, *NDMM* newly diagnosed multiple myeloma, *OS* overall survival, *PFS* progression-free survival, *SWOG* Southwest Oncology Group, *VGPR* very good partial response

## The impact of disease and patient heterogeneity on clinical outcomes

A substantial challenge in cross-study comparisons and in comparisons between clinical trials and real-world data is disease and patient heterogeneity, which may influence treatment outcomes^31,32^. Differences in eligibility criteria across clinical trials can result in imbalances between trial populations that can affect outcomes, as exemplified by the range of outcomes seen with the Rd ( ± placebo) comparator arms in phase 3 trials in RRMM (Table 1)^15,17,19,20^. Differences were seen in response rates and outcomes, with median PFS ranging from 14.9 months in ELOQUENT-2 to 18.4 months in POLLUX.

Similarly, real-world populations may have substantially different patient/disease characteristics compared with clinical trial cohorts, and thus, clinical trial outcomes may not reflect real-world experience. In a recent analysis of the CONNECT-MM registry, it was reported that up to 40% of patients treated in routine care would be ineligible for enrollment to randomized controlled trials in newly diagnosed MM due to common stringent eligibility criteria^33^. Importantly, this analysis showed that trial-ineligible patients had a significantly lower 3-year survival rate (63%) compared with trial-eligible patients (70%, *p*-value = 0.0392)^33^. Broadening eligibility criteria to increase the generalizability of clinical trial results is a recognized need towards more informed treatment decision-making^33,34^.

There are a range of prognostic patient and disease characteristics that may vary between clinical trial populations and between clinical trial and real-world cohorts, potentially driving differences in outcomes. These are reviewed below.

### Geographic variations

Geographic differences in patient populations and in treatment availability may affect outcomes. In addition to the potential for drug metabolism/pharmacology to be affected by ethnic differences^35^, clinical presentation may differ between regions. For example, Chinese MM patients have much more advanced-stage disease at diagnosis compared with Western MM patients^36^. Furthermore, regional variation in therapies that patients are exposed to prior to and after trial interventions may also impact outcomes in intention-to-treat analyses of protocol therapy. For example, the use of melphalan–prednisone-based regimens is common in Europe^37,38^ but rare in North America, where the majority receive Rd in the non-transplant setting^4^. Long-term outcomes such as OS will also depend on the number of available subsequent active treatment options in a particular country or region. Given these differences, real-world outcomes with a specific regimen in a particular region may not reflect outcomes from clinical trials conducted in a geographically broader or different patient population. These aspects highlight the importance of considering the regional composition of patient populations when evaluating different clinical trial or real-world data.

### Age and comorbidities

Patient age is a key predictive parameter in MM, with median OS decreasing from 6.4 years in patients aged < 50 years to 2.5 years in patients aged ≥ 80 years in one analysis of OS by age cohort^1,39^. The presence and extent of comorbidities are also prognostically important^40–43^, with greater comorbidities and poorer performance status resulting in worse responses to treatment; however, these are rarely reported in trials. Comorbidities such as diabetes (21.5% vs. 16.3%), cardiovascular disease (including hypertension, 62.0% vs. 52.7%), and some of the CRAB criteria for MM–hypercalcemia (11.0% vs. 5.5%), renal impairment (38.9% vs. 6.2%), and anemia (59.5% vs. 39.5%)–have been shown to be more common in clinical trial-ineligible versus clinical trial-eligible patients in the CONNECT-MM registry, further contributing to the gap between efficacy and effectiveness^33^.

### Disease stage

International Staging System (ISS) stage is widely reported in MM clinical trials and frequently used as a stratification factor^20^ to avoid imbalances between study arms, as it has important prognostic implications. However, the relative proportions of patients with stage I, II, and III disease may vary substantially across clinical studies and may be reported at different time points, i.e., either at initial diagnosis or at study entry. For example, in the CASTOR phase 3 study of daratumumab plus bortezomib-dexamethasone in RRMM, 39%, 37.5%, and 23.5% of patients on the investigational arm had stage I, II, and III disease at screening, respectively^16^, whereas in the TOURMALINE-MM1 phase 3 study of ixazomib-lenalidomide-dexamethasone in RRMM the respective proportions were 64%, 24%, and 12%^20^. In contrast, disease staging in the ASPIRE phase 3 study of carfilzomib-lenalidomide-dexamethasone in RRMM was reported from initial MM diagnosis, at which time 17.4%, 24.4%, and 43.7% of patients had stage I, II, and III disease, respectively, with 14.5% unknown^19^.

Similarly, the relative proportions may vary substantially between clinical studies and real-world patient populations; in the analysis of patients in the CONNECT-MM registry, 29%/27%/22% of clinical trial-eligible patients had stage I/II/III disease, compared to 13%/22%/40% of patients ineligible for clinical trials, suggesting that real-world populations may include a greater proportion of patients with advanced-stage disease^33^. Such a discrepancy may result in poorer outcomes among real-world versus clinical trial populations.

More recently, the ISS has been updated to incorporate the presence of cytogenetic abnormalities and lactate dehydrogenase levels as additional prognostic factors^32^. Although it is suggested that the Revised-ISS is a more powerful prognostic system than the original ISS classification system, it has not been substantially implemented to date in MM; therefore, these data are not currently widely reported in clinical trials or in real-world analyses, adding another source of potential unobserved confounding.

### Disease subtype

A small proportion ( < 3%) of MM patients have non-secretory disease^44^, which may have a variable prognosis^44^, whereas others have disease that is evaluable according to free light chain (FLC) levels/ratio only. Furthermore, the variability in FLC levels/ratio in patients with non-secretory MM^45^ and differences in FLC/light chain development during follow-up in non-secretory patients^46^ may result in challenges for interpretation of response and progression. Serum FLC ratio is prognostic for PFS and OS in MM, but discrepancies have been reported in FLC-based relapse versus conventional relapse, with serum FLC escape occurring a median of 3.8 months earlier than conventional relapse in 20% of patients in one retrospective investigation^47^. Thus, there is a potential impact on the interpretation of overall outcomes between studies that include or exclude FLC-only evaluable patients.

### Renal impairment

Renal impairment is a known adverse prognostic indicator and a frequently applied exclusion criterion in clinical trials; however, as many as 59% of RRMM patients treated in routine care have a history of renal impairment (defined by ICD-9 diagnosis code, creatinine clearance < 40 mL/min, or serum creatinine > 2 mg/dL) at the time they initiate salvage therapy^48^. The exclusion criterion for creatinine clearance is commonly defined in clinical trial eligibility criteria as < 30 mL/min but has also been defined as < 20, < 30, or < 50 mL/min in phase 3 trials in RRMM;^16,19,20^ reported rates of renal impairment in phase 3 trials include 7–20% of patients with creatinine clearance < 50 mL/min^18,19^ and 24–27% with creatinine clearance < 60 mL/min^16,17,20^. Such differences in eligibility criteria and patient populations can impact outcomes and the translation of findings to the real-world setting.

### Cytogenetic abnormalities

There is substantial genetic complexity in MM, and multiple primary and secondary cytogenetic abnormalities can potentially impact outcomes^31,49^. Not all of the abnormalities now recognized as conferring poor prognosis are routinely collected or reported in clinical trials or observational studies, including deletion 17 [del(17)], the translocations t(4;14), and t(14;16)^19,20^, 1q amplification^50^, and 1p deletion^51^. The proportion of patients with cytogenetics reported is often low in clinical trials (e.g., 47% of patients in FIRST;^9^ 53% in ASPIRE^52^), leading to a high level of missing information, although this proportion was higher in the TOURMALINE-MM1 (76%)^20^, CASTOR (71%)^16^, and POLLUX (77%)^15^ trials. Similarly, these data appear to be limited in analyses of routine practice, with conventional cytogenetic and fluorescence in situ hybridization (FISH) analysis being reported in only 37–63.2% of patients in recent real-world analyses;^24,53,54^ a contributory factor may be the limited availability of these analytical techniques, notably FISH, at different sites around the world. This raises the possibility of ‘hidden’ imbalances in prognostic markers across these studies, making indirect comparisons even more difficult. Additionally, there is no consistent methodology (and therefore sensitivity) or cut-off to define the presence of a high-risk abnormality; for example, presence of del(17p) has variously been defined in recent clinical studies based on abnormalities in a single cell^17^, through 1.5–7.5% of cells^20^, up to 60% of cells^19^. Yet, different clone sizes may have different prognostic impact, and thus patient populations appearing to have the same rate of a specific cytogenetic abnormality may have different outcomes.

### Prior treatment exposure

Prior therapy exposure can markedly affect treatment outcomes. Refractoriness to previous therapy^55^ and number and type of previous lines of therapy^56^ are known determinants of response to subsequent therapy; however, these may be confounded by the use of different definitions for determining ‘refractory’ disease and classifying a line of therapy between clinical trials and compared with the real-world setting. Patients typically have poorer outcomes with successive lines of therapy, including shorter disease-free intervals or periods of disease control, both in clinical trials and in the real-world setting^24,38,57^, associated with increasing disease refractoriness and accumulating frailty/comorbidities. Standard clinical trial eligibility criteria typically exclude more heavily pretreated patients^38^. Additionally, type of prior therapy can impact response to treatment. For example, in North America, many patients receive Rd^4^ initially and therefore may develop lenalidomide-refractory disease. However, phase 3 clinical trials of regimens consisting of an Rd backbone have often excluded lenalidomide-refractory patients^15,17,20^, making it difficult to translate the efficacy of the studied regimen to effectiveness in a real-world patient population.

## The potential impact of clinical trial design

An important consideration in the interpretation of clinical trial results is the study design, specifically whether an open-label or placebo-controlled double-blind design was used. An unblinded randomized trial design may introduce bias when using PFS as an endpoint^58^, leading to possible differential withdrawal rates associated with more patients on the control arm withdrawing from the study for reasons other than disease progression and potentially receiving a subsequent therapy without having disease progression. These issues may be overcome through the use of central blinded review^58^. Similarly, there is the potential for bias in quality of life (QoL) and patient-reported outcome endpoints in unblinded studies, associated with a more ‘upbeat’ evaluation of their status by patients enrolled to receive the novel, ‘exciting’ treatment option^59^.

## Interpreting endpoint data in multiple myeloma clinical trials

Discrepancies occur between clinical trials with regards to criteria used for response assessment and schedule of assessments, potentially impacting the interpretation of response and/or progression data. The International Myeloma Working Group (IMWG) has recently published updated consensus criteria for assessment of response and progression;^60,61^ however, older studies are more likely to have used the European Group for Blood and Marrow Transplantation (EBMT) criteria^62^ or the original IMWG uniform response criteria^63^. Therefore, PFS may be affected by the stringency of the criteria used for determining relapse from CR; this so-called ‘CR penalty’ when using the EBMT criteria^64^ arises because relapse from CR is defined as M-protein reappearance on immunofixation^62^, whereas for PFS evaluation, the IMWG criteria require an absolute increase in serum or urine M-protein to define relapse from CR^63^. Furthermore, response and progression assessment may differ in the real-world setting, with potentially less rigorous criteria used to determine progression and to evaluate response than in a clinical trial. For example, bone marrow confirmation and flow cytometry/PCR assessment of minimal residual disease (MRD) may not routinely be conducted or repeated for cases of suspected CR/stringent CR. Additionally, depending on the rigor of response assessment and extent of available documentation, all subcategories of depth of response may not feasibly be reported in the real-world setting.

While it is generally established that, with all other things being equal, depth of response is clearly associated with improved long-term outcomes^65^, there are examples of clinical trial data in which the response rates do not correlate with PFS and/or OS^37,66–69^. These discordances may arise for a number of reasons, such as the effects of toxicity (e.g., treatment discontinuation, death) or more aggressive relapse, and should be considered when interpreting clinical trial data. In a phase 3 study of lenalidomide plus high-dose versus low-dose dexamethasone in NDMM^66^, the response rate was higher (79% vs. 68%) in the high-dose dexamethasone arm, but due to toxicities associated with the higher dose of dexamethasone, the 1-year OS rate was lower (87% vs. 96%). Similarly, in a randomized study of thalidomide-dexamethasone (TD) versus melphalan-prednisone (MP) as frontline therapy in transplant-ineligible NDMM patients^69^, response rates were higher with TD versus MP (68% vs. 50%; CR + VGPR: 26% vs. 13%) but median OS was significantly shorter (41.5 vs. 49.4 months). This was also seen in the phase 3 FOCUS study of single-agent carfilzomib versus best supportive care in heavily pretreated RRMM patients; carfilzomib resulted in a higher response rate (19% vs. 11%), but this did not translate into improved outcomes in these ‘end-stage’ patients, in whom survival times would be expected to be limited–median PFS (3.7 vs. 3.3 months) and OS (10.2 vs. 10.0 months) were similar between arms^67^. Additionally, in the phase 3 CLARION study of KMP versus VMP in NDMM, the response rate was slightly higher with KMP (84% vs. 79%, including 26% vs. 23% CR) but PFS was similar (median 22.3 vs. 22.1 months)^68^.

A more important prognostic factor for improved outcomes in clinical trials may be achievement of a sustained response^70^ or the improvement of response over the course of treatment^71^. Evaluation of best response alone may not capture evolution of response and the impact of late, evolving responses on outcomes^72^, nor does it capture short-lived responses, which reflect aggressive disease. These aspects may account for discordance between response rates and long-term outcomes. Additionally, standard depth-of-response assessments may potentially mask responses of greater depth in both clinical trials and the real world. For example, while CR is associated with improved PFS and OS in MM^73^, this standard category of depth of response may include patients with differing levels of MRD. Increasing depth of MRD elimination has been associated with improvements in outcomes;^74^ thus, not all CRs are equal, and an imbalance in the ‘hidden’ rate of MRD elimination may confound interpretation of clinical trial and real-world data. In the GEM2005 phase 3 study in transplant-ineligible NDMM patients of VMP versus VTP induction plus VT vs. VP maintenance, although the CR rate favored the VTP arm (20% vs. 28%), among patients achieving CR on VMP and VTP, 70% and 45%, respectively, were MRD-negative by multiparameter flow cytometry, which translated into longer median PFS (32 vs. 23 months) and median OS (63 vs. 43 months) in the VMP arm^37^. This imbalance in MRD elimination may have contributed to the apparent disconnect between the relative CR rates and PFS/OS with VMP and VTP.

Furthermore, cross-trial comparison of MRD-negative rates may also be confounded by the use of different techniques, with different sensitivities, for determining MRD elimination. Of note, the limited use of MRD negativity as a treatment goal in clinical practice may result in poorer outcomes compared to a clinical trial, with real-world patients potentially discontinuing treatment upon having achieved a CR but before obtaining possible MRD elimination.

### Patient-reported outcomes

Patient-reported outcomes are also of importance in MM, particularly in the context of long-term or continuous therapy administration, which is made feasible if therapies have minimal impact on patients’ QoL^75,76^. MM patients typically have a high symptom burden and impaired QoL^76^, and health-related QoL questionnaires have been employed with the aim of determining the extent of disease-related effects and the impact of treatment response and toxicity on QoL^75^. Recent novel therapies for RRMM have demonstrated a limited adverse impact or a positive impact on patients’ QoL in phase 3 trials, likely associated with a reduction in disease symptoms^20,75,77–79^. However, in interpreting these findings, it is important to consider the study design–as QoL data are susceptible to bias in open-label studies. Additionally, the instrument(s) employed should be evaluated; prior to the introduction of more recent instruments, there was a historical lack of an MM-specific QoL questionnaire to reflect the key aspects of disease and treatment burden on patients in MM;^80^ thus, while improvements may be recorded in more generic instruments, these may not capture some MM-specific issues of importance to patients. Furthermore, current instruments may not be sufficiently sensitive to detect QoL variations, depending on the type of treatment, particularly effects specific to recent novel agents. Although these tools are validated and widely used, they may lack the power to reflect the details of the real impacts of treatment on QoL^81^.

The timing of the patient-reported outcome assessments may also influence the extent to which effects of treatment toxicity are adequately captured, with patient-reported outcomes from clinical studies typically collected over a relatively short follow-up period and at the beginning of a treatment cycle, thus only reflecting patient states with resolved adverse events and/or short-term QoL information^82^. Intra- and inter-trial differential attrition and compliance rates across comparator arms may contribute to data missing not at random that may bias the relative treatment effects. Furthermore, there is often variation across trials in the types of analytic methods applied, including non-uniform approaches that address missing data, which may have a substantial impact on the point estimates of treatment effects. Patient-reported outcomes should thus be interpreted in this context.

## Understanding disparities between different clinical trials and between real-world experience: acknowledging real-world considerations

### Targeted literature review: real-world and clinical trial data in RRMM

There are inherent difficulties in obtaining and analyzing complete sets of real-world data. Several methods can be used, including prospectively designed observational studies, retrospective chart reviews, and claims database analyses. However, currently there are no standardized methods for claims-based outcomes research in MM. The varied application of MM treatment algorithms across studies can also present challenges in retrospective interpretation of electronic medical record (EMR) analyses and other real-world data, for example, how to determine lines of therapy or when a patient is receiving maintenance rather than extended induction therapy.

To investigate discrepancies between clinical trial efficacy and real-world effectiveness, we conducted a targeted literature review to identify sources of real-world, non-clinical-trial data in RRMM and evaluated these data in the context of phase 3 clinical trial results. For a description of the methodology, please see the supplementary information. Data from 61 relevant publications and abstracts are summarized in Supplementary Tables S1 and S2, and corresponding data from phase 3 studies are summarized in Supplementary Table S3.

Tables 2 and 3 present summaries of the PFS/time to next therapy (TTNT) and duration of therapy (DOT) data from relevant real-world reports and clinical studies in RRMM patients with 1–3 prior therapies. Our findings show that outcomes seen in clinical trials are not always replicated in the real-world setting. The ranges of median PFS/TTNT values in real-world reports were generally shorter than those reported in phase 3 clinical studies (Table 2), with a larger gap seen with injectable PI-immunomodulatory drug-based triplet regimens. Conversely, PFS/TTNT in clinical and real-world studies appeared more closely aligned with all-oral regimens. As noted, longer DOT has been associated with prolonged PFS/OS^9,83,84^. Therefore, these data were evaluated similarly to PFS/TTNT data (Table 3). The ranges of median DOT values in the real-world reports were shorter than, or similar to, those reported in phase 3 clinical studies in RRMM patients with 1–3 prior therapies. Although reasons for discontinuations were not consistently reported, treatment toxicity is anticipated to have contributed to patients discontinuing therapy, and thus tolerability may be an important factor for a number of regimens in the real-world setting. In a real-world analysis of treatment among US community oncology practices, rates of discontinuation due to toxicity for second-line and third-line regimens ranged from 15.3−32.0%;^24^ this contrasts with rates of 6.7%–20.9% in recent phase 3 studies in RRMM after typically 1–3 prior lines^15–20^. Of note, in our analysis, the ranges of median PFS/TTNT and DOT with bortezomib-based regimens appeared similar in clinical trials and real-world analyses. This might possibly be associated with the fixed duration of therapy utilized in bortezomib phase 3 studies closely mirroring the length of therapy tolerated in the real world.

**Table 2:** Comparison of PFS/TTNT from real-world reports and phase 3 clinical studies in RRMM patients after 1–3 prior lines of therapy; see Supplementary Table S4 for full details of studies cited for each range/piece of data

Regimens	Phase 3 clinical studies	Real-world reports		
		All reports identified	Studies/registry analyses^a^	EMR/chart review/claims analyses^b^
All regimens combined	Not applicable	6–15.1	6.4–14.1	6–15.1
PI doublet / PI-based^c^	Btz: 6.2–9.4 Cfz: 14.9–22.2	Btz: 5.7–11.9 Cfz: 3.2–10.6	Btz: 5.7–11.3 Cfz: 5.6–10.6	Btz: 6.9–11.9 Cfz: 3.2–9.4
PI-alkylator triplet	12–18.4^d^	6.5–16.2	NR	6.5–16.2
Injectable PI-immunomodulatory drug triplet	18.3–29.6	9.4–25.3	NR	9.4–25.3
Oral PI-immunomodulatory drug triplet	17.5–20.6	19.2–27.6	27.6	19.2
Len doublet / len-based^c^	11.1–18.4	6.6–21	6.6–8.7	7–21

*Note*: Data shown are ranges of median PFS/TTNT (months) reported from multiple studies/analyses

^a^Including prospective and retrospective registry studies and observational studies, and analyses of data from named patient programs/compassionate use programs

^b^Including single-center, retrospective chart reviews, EMR reviews, longitudinal chart reviews

^c^Regimen not specified beyond ‘PI-based’ or ‘len-based’ in some real-world reports

^d^Data from two phase 2 studies of VCd

**Table 3:** Comparison of DOT from real-world reports and phase 3 clinical studies in RRMM patients after 1–3 prior lines of therapy; see Supplementary Table S5 for full details of studies cited for each range/piece of data

Regimens	Phase 3 clinical studies	Real-world reports		
		All reports identified	Studies/registry analyses^a^	EMR/chart review analyses^b^
All regimens combined	Not applicable	2.1–7	4.6	2.1–7
PI doublet / PI-based^c^	Btz: 4–6.2 Cfz: 9.2	Btz: 3.7–6.6 Cfz: 2.3–4.6	Btz: 4.1–4.5 Cfz: 3.4	Btz: 3.7–6.6 Cfz: 2.3–4.6
PI-alkylator triplet	NR^d^	5	NR	5
Injectable PI-immunomodulatory drug triplet	5.2–20.3	3.4–8.7	5	3.4–8.7
Oral PI-immunomodulatory drug triplet	15.7	5.5–7.2	5.5^e^–7.2	5.5
Len doublet/len-based^c^	10.1–13.8	3.1–16.8	4.9–5.6	3.1–16.8

*Note*: Data shown are ranges of median DOT (in months) reported from multiple studies/analyses

^a^Including prospective and retrospective registry studies and observational studies, and analyses of data from named patient programs/compassionate use programs

^b^Including single-center, retrospective chart reviews, EMR reviews, longitudinal chart reviews

^c^Regimen not specified beyond ‘PI-based’ or ‘len-based’ or ‘IMiD-based’ in some real-world reports

^d^Data from two phase 2 studies of VCd

^e^Cycles (28-day)

### Reasons for discrepancies between real-world and clinical trial data

Patient selection is a known contributor to the gap between efficacy in clinical trials and effectiveness in the real-world setting^85^. Only 3% of patients participate in oncology trials in the United States^86^, while the UK is at the other end of the spectrum with 35% of MM patients participating in research^87^. Older patients, those with higher comorbidity burden, and patients from lower socioeconomic background are under-represented in trials^88^. In MM in particular, advanced age, functional decline, and comorbid conditions represent components of frailty that are predictive of mortality and toxicity risk^43^. As discussed earlier, the disparities seen between real-world effectiveness and clinical trial efficacy may, in part, be associated with differences between eligibility criteria and characteristics of study populations. This makes it critical to compare the baseline patient characteristics when interpreting results of studies. It is interesting to note that the gap between routine clinical practice and clinical trial outcomes in later lines of therapy (i.e., in a more heavily pretreated/refractory population) appears smaller than in earlier lines of therapy for RRMM. It may be speculated that this could be due to closer alignment between the real-world and clinical trial patient populations in this setting or that the generally limited benefit in this setting results in more aligned outcomes.

Other potential sources for the gap between DOT achieved in the real-world versus the clinical trial setting may include treatment center effect (academic vs. community centers having differing levels of experience of managing patients being treated with novel regimens, with community centers being under-represented in clinical trials), study design (e.g., use of treat-to-progression clinical study designs not being feasible or not being utilized in the real-world setting), and physician and patient preference. Furthermore, protocol-directed treatment rigor in clinical trials dictates dose modifications that may lead to better tolerability and longer duration of therapy in clinical trials. This is important because, as demonstrated in a number of clinical studies, a fixed duration of therapy may be associated with poorer outcomes in real-world practice, e.g., if patients discontinue therapy due to poor tolerability or high burden of treatment not seen or anticipated in the clinical trial^84,89^. Similarly, multiple unplanned dose modifications outside of the clinical trial setting, such as dose reductions, treatment delays, or dropping a component of therapy may adversely impact the relative dose intensity of active drugs, which has been associated with poorer OS^90^.

Real-world considerations such as the tolerability, convenience, and practicality of therapy are not captured within clinical trial reports or using conventional endpoints; however, they may also contribute to discrepancies between clinical trial and real-world outcomes. For example, treatment with a novel agent or regimen in the real-world setting may be associated with substantial patient time and economic burden^91,92^, including direct and indirect costs associated with medications and attending regular clinic visits. The burden of treatment, such as the route of administration and travel to the treatment center^10,93^, may not be captured by standard clinical trial endpoints but may substantially affect the feasibility of long-term treatment in the real-world setting as patients may succumb to treatment fatigue outside of the ‘motivating’ environment of clinical trial participation. Differences in adherence to treatment between a stringently monitored clinical trial setting and routine care may thus impact the generalizability of trial results to the real world.

Similarly, as noted earlier when discussing patient-reported outcomes, clinical trial data may not necessarily capture the cumulative long-term burden of the disease, its treatment, and the associated comorbidities and toxicity, or the psychosocial effects and lifestyle impact of living with MM^82^. These considerations must be acknowledged in tandem with clinical trial efficacy and safety data when interpreting data on different regimens; in this context, it is also important for patients to be well educated about treatment options and for clinicians to consider patient goals of therapy for what remains a generally incurable disease. For example, while a highly active and aggressive treatment approach, involving considerable burden and possibly toxicity, may be acceptable for younger, fitter patients if it offers the potential of long-term remission and elimination of MRD, for other groups of patients, the achievement of long-term disease control with minimal toxicity and preserved QoL may be an equally meaningful endpoint.

In consideration of these issues, a number of recommendations may be made to aid the broader real-world use of drugs and attempt to close the efficacy versus effectiveness gap. First, greater education of academic and community physicians can provide them access to clinical trial management algorithms to improve utilization of novel agents and regimens. Associated guidance on side-effect management, QoL monitoring, and support regarding adherence to medication should be provided to enable prolonged therapy. Second, ensuring drug labels are updated for use in routine practice following approval of a novel agent in combination with an existing agent is recommended, as clinicians who are utilizing drugs per label need up-to-date guidance on the use of these drugs in all approved indications. Indications for use will need to be updated/maintained regularly to help ensure optimal use of therapy. Furthermore, the use of adaptive study designs, including for regulatory pathway studies, that could be adjusted to reflect changes in the treatment landscape would help with providing relevant data to the current real-world setting once results become available^94,95^.

## Conclusions

In conclusion, interpretation of data across clinical studies in MM and between clinical studies and real-world analyses is highly complex, with a multitude of factors confounding simple interpretation of efficacy, safety, and effectiveness between regimens. Both clinical trials and observational studies provide complementary information of importance in treatment decision-making. Clinical trials isolate treatment efficacy in selected patient populations and are less prone to bias, with good internal validity, while observational studies provide insights into treatment effectiveness in heterogeneous patient populations. In the absence of head-to-head comparisons between regimens, indirect comparisons of clinical study findings should be made with extreme caution; the most valid approach may be indirect comparisons of relative efficacy versus a common comparator using hazard ratios. Equally importantly, clinicians need to consider patient-related factors that may impact the translation of clinical trial outcomes to daily practice, such as QoL, tolerability, and burden of treatment, which may also help with tailoring treatment approaches for individual patients and thus optimize outcomes.

In the future, it will be important not only to systematically assess the discrepancies between clinical trials but also the inconsistencies between the real-world and clinical trial settings, and to evaluate in greater detail the drivers of these differences. This may be supported by increased utilization of patient-reported outcomes, which are of increasing importance in informing treatment. Real-world effectiveness should be a metric considered in routine clinical practice, as it will be important to develop drugs and combinations that will be effective in the real world across patient populations, outside of the rigorously controlled clinical trial setting.

## Electronic supplementary material

Supplement - clean
