# Supplementary material for: Interpreting clinical trial data in multiple myeloma: translating findings to the real-world setting
Source: Blood Cancer J. 2018 Nov 9;8(11):109. doi: 10.1038/s41408-018-0141-0 (PMC6226527; doi:10.1038/s41408-018-0141-0)
Supplement: Supplementary file 1 — Supplement - clean [file 41408_2018_141_MOESM1_ESM.docx]

**Interpreting clinical trial data in multiple myeloma: translating findings to the real-world setting**

**Authors:** Paul G. Richardson, Jesus F. San Miguel, Philippe Moreau, Roman Hajek, Meletios A. Dimopoulos, Jacob P. Laubach, Antonio Palumbo, Katarina Luptakova, Dorothy Romanus, Tomas Skacel, Shaji K. Kumar, Kenneth C. Anderson

**Supplementary information**

***Targeted literature review: real-world and clinical trial data in RRMM – methodology***

To investigate discrepancies between clinical trial efficacy and real-world effectiveness, we conducted a targeted literature review to identify sources of real-world, non-clinical-trial data in relapsed/refractory multiple myeloma (RRMM) and evaluated these data in the context of phase 3 clinical trial results for recently approved and commonly used regimens. This review comprised a PubMed search to identify publications containing relevant real-world data that were published in the past 10 years; search terms used were ‘myeloma’ plus ‘real-world’, ‘electronic medical record’/‘EMR’, ‘community’, or ‘registry’. This search identified 959 records of possible relevance, of which 913 records were discarded based upon review of title and/or abstract. The full text of 46 records was subsequently reviewed to identify papers that contained real-world information on treatment duration and/or outcomes in patients with RRMM, either overall or for specific regimens; 12 relevant publications were thereby manually selected.^1-13^ A similar search was also conducted of the abstracts from the past 3 annual meetings of the American Society of Hematology (ASH; 2015–2017), the American Society of Clinical Oncology (ASCO; 2015–2017), and the European Hematology Association (EHA; 2015–2017), and from the past 2 International Myeloma Workshop (IMW) meetings (2015, 2017); 49 relevant abstracts were identified.^14-62^ Data from the 61 identified publications and abstracts are summarized in Tables 2 and 3 (and Supplementary Tables S1 and S2), and corresponding data from recent phase 3 studies in RRMM are summarized in Table 4 (and Supplementary Table S3). Supplementary Tables S4 and S5 provide referenced summaries of progression-free survival (PFS)/time to next therapy (TTNT), and duration of treatment (DOT), mirroring Tables 5 and 6 in the main manuscript.

For the purposes of comparison of real-world data with recent clinical trial data in RRMM, data were extracted from reports in RRMM patients with 1–3 prior therapies, given the known adverse prognostic impact of multiple prior treatments and prior exposure/refractoriness to specific agents. Furthermore, to align with regimens available in routine clinical practice, and thus the availability of corresponding real-world data, only proteasome inhibitor−based and immunomodulatory drug-based regimens were included. Due to the inherent difficulty of obtaining precise progression dates in claims-based, electronic medical record-based outcomes research and in some retrospective chart reviews, TTNT was used as proxy for PFS where required and available. Real-world reports included population-based analyses encompassing a range of regimens in routine clinical practice as well as observational studies reporting outcomes for regimen-specific therapy. Additionally, real-world reports comprised different levels of evidence based on the different data sources utilized, including population-representative studies, registry analyses, and retrospective EMR/chart review and administrative claims analyses. The real-world findings have been reported separately according to these different types of study.

**Supplementary tables**

**Supplementary Table S1:** Publications reporting real-world data on treatment duration and/or outcomes in patients with RRMM, with full study citations

| Study | Regimen | Prior lines | N | Age* | ISS I / II / III, % | HR cyto, % | RI, % | DOT,* mos | PFS/TTNT,* mos | TTP,* mos | OS,* mos |
| --- | --- | --- | --- | --- | --- | --- | --- | --- | --- | --- | --- |
| Spanish compassionate use registry^4^ | Rd | 3* | 111 | 66.5 | NR | 17.1 | 12.6 | 4.9 | NR | 13.0 | 17.4 |
| US claims database analysis^5^ | Pom-based | NR | 264 | 62 | NR | NR | 6.4 | 5.9 | 11.9 | NR | NR |
|  | Cfz-based | NR | 190 | 61 | NR | NR | 14.2 | 4.2 | 9.4 | NR | NR |
| US community oncology study^1^ | All | 1 | 391 | Mean 68.2 | 19.6 / 33.5 / 49.6 | 16.6 | 34 | 4.2 | 10.5 | NR | NR |
|  |  | 2 | 391 | NR | NR | NR | NR | 3.1 | 7.9 | NR | NR |
|  |  | 3 | 229 | NR | NR | NR | NR | 2.1 | 7.2 | NR | NR |
|  | Btz-based | 1 | 166 | NR | NR | NR | NR | 3.8 | 9.8 | NR | NR |
|  |  | 2 | 128 | NR | NR | NR | NR | 3.7 | 9.1 | NR | NR |
|  |  | 3 | 73 | NR | NR | NR | NR | NR | 6.9 | NR | NR |
|  | Cfz-based | 1 | 6 | NR | NR | NR | NR | 2.7 | 3.2 | NR | NR |
|  |  | 2 | 37 | NR | NR | NR | NR | 2.4 | 3.6 | NR | NR |
|  |  | 3 | 46 | NR | NR | NR | NR | NR | 7.2 | NR | NR |
|  | Len-based | 1 | 103 | NR | NR | NR | NR | 5.6 | 13.2 | NR | NR |
|  |  | 2 | 88 | NR | NR | NR | NR | 3.1 | 7 | NR | NR |
|  |  | 3 | 30 | NR | NR | NR | NR | NR | 9.8 | NR | NR |
|  | Pom-based | 1 | 4 | NR | NR | NR | NR | 3.8 | 5.7 | NR | NR |
|  |  | 2 | 17 | NR | NR | NR | NR | 1.4 | 9.6 | NR | NR |
|  |  | 3 | 12 | NR | NR | NR | NR | NR | 3.8 | NR | NR |
|  | PI-len/thal-based | 1 | 60 | NR | NR | NR | NR | 3.6 | 12.7 | NR | NR |
|  |  | 2 | 59 | NR | NR | NR | NR | 3.4 | 9.4 | NR | NR |
|  |  | 3 | 26 | NR | NR | NR | NR | NR | 11.0 | NR | NR |
| Greek Myeloma Study Group analysis^6^ | Rd | 2* | 212 | Mean 68 | 50.8 / 29.4 / 19.8 | 19.6 | NR | 16.8 | NR | NR | NR |
| UK multicenter retrospective analysis^7^ | Pom-dex | 3* | 85 | 66 | 9.4 / 15.3 / 18.8 | 44.4 | 22.4 | 4 cycles (28 days) | 5.2 | NR | 13.7 |
| Italian multicenter retrospective analysis^3^ | Rd | 1–2 | 290 | 70 | NR | NR | 13 | 7 cycles (28 days) | NR | 11 | NR |
| Canadian single-center retrospective analysis^8^ | CyBorP/D | 2* | 96 | 64 | NR | 23 | NR | 5 cycles (28 days) | 16.2 | NR | 26.3 |
| UK single-center retrospective analysis^9^ | Btz- based retreatment | 1 | 23 | 64 | 48 / 26 / 26 | 22 | NR | 5 cycles (NR) | NR | 14.4 | NR |
| US EMR database analysis^10^ | All | 3* | 500 / 162 | 70 / 67 | NR | NR | NR | NR | NR | NR | 7.9 |
| European retrospective chart review^2^ | All | 1 | 1380 | NR | 16 / 35 / 49 | NR | 20 | 7 | 12 | 13 | NR |
|  |  | 2 | 1815 | NR | NR | NR | NR | 6 | 9 | 7 | NR |
|  |  | 3 | NR | NR | NR | NR | NR | 5 | 6 | 5 | NR |
| Australian retrospective chart review^11^ | Pom-dex | 5 | 87 | 65 | NR / NR / 53 | NR | 14 | 3 | 3.4 | NR | 7.5 |
| Kansai Myeloma Forum analysis^13^ | Pom-dex | 4 | 108 | 69 | 27 / 35 / 32 | NR | NR | NR | 4.4 | NR | Not reached |

*Median, except where stated.

Btz, bortezomib; CCI, Charlson Comorbidity Index score; Cfz, carfilzomib; dex, dexamethasone; cyto, cytogenetics; DOT, duration of therapy; EMR, electronic medical record; HR, high-risk; IMiD, immunomodulatory drug; ISS, International Staging System; Len, lenalidomide; NR, not reported; OS, overall survival; PFS, progression-free survival; PI, proteasome inhibitor; Pom, pomalidomide; Rd, lenalidomide, dexamethasone; RI, renal impairment or renal failure; Thal, thalidomide; TTNT, time to next therapy; TTP, time to progression.

**Supplementary Table S2:** Abstracts reporting real-world data on treatment duration and/or outcomes in patients with RRMM, with full study citations; the increasing numbers of reports at more recent congresses potentially reflects a growing recognition of the importance of these data

| Study | Regimen | Prior lines | N | Age* | ISS I / II / III, % | HR cyto, % | RI, % | DOT,* mos | PFS/TTNT,* mos | TTP,* mos | OS,* mos |
| --- | --- | --- | --- | --- | --- | --- | --- | --- | --- | --- | --- |
| ASH 2017 |  |  |  |  |  |  |  |  |  |  |  |
| Observational study^50^ | All | 2 | 337 | 67 | NR | NR | NR | 4.6 | NR | NR | NR |
|  | Dara-based | 3 | 26 | 66 | NR | NR | NR | 4.2 | NR | NR | NR |
|  | IMiD-based | 2 | 75 | 65 | NR | NR | NR | 5.6 | NR | NR | NR |
|  | PI-based | 2 | 174 | 68 | NR | NR | NR | 4.1 | NR | NR | NR |
|  | PI-IMiD-based | 2 | 62 | 64 | NR | NR | NR | 5 | NR | NR | NR |
| US Oncology database analysis^51^ | Cfz-based | 1–4 | 718 | NR | 21/25/34 | NR | 11.3 | NR | 10.6 | NR | NR |
| Czech Specific Healthcare Program^52^ | Dara | 4.5 | 14 | 61 | NR | NR | NR | NR | 4.6 | NR | 7.2 |
| US national EMR analysis^53^ | Pom-based | 1 | 567 (LOT) | 66 | NR | NR | 9 | 1.5 | 4.2 | NR | NR |
|  |  | ≥2 | 567 (LOT) | 66 | NR | NR | 9 | 2.3 | 4.6 | NR | NR |
|  | Cfz-based | 1 | 609 (LOT) | 65 | NR | NR | 11.3 | 2.3 | 6.2 | NR | NR |
|  |  | ≥2 | 609 (LOT) | 65 | NR | NR | 11.3 | 2.4 | 4.7 | NR | NR |
|  | Ixazomib-based | 1 | 172 (LOT) | 65 | NR | NR | 14.5 | 1.4 | 2.9 | NR | NR |
|  |  | ≥2 | 172 (LOT) | 65 | NR | NR | 14.5 | 2 | 3.5 | NR | NR |
|  | Elotuzumab-based | 1 | 76 (LOT) | 64 | NR | NR | 15.8 | 4.4 | 6.5 | NR | NR |
|  |  | ≥2 | 76 (LOT) | 64 | NR | NR | 15.8 | 2.7 | 3.7 | NR | NR |
|  | Dara-based | 1 | 321 (LOT) | 65 | NR | NR | 10.3 | 1.8 | 3.2 | NR | NR |
|  |  | ≥2 | 321 (LOT) | 65 | NR | NR | 10.3 | 2.1 | 2.8 | NR | NR |
| Hungarian named patient program^54^ | Ixazomib-Rd | 2 | 77 | NR | NR | 37 | NR | 5.5 cycles (28-day) | 46% at 12-months | NR | NR |
| Greek, UK, Czech named patient program^55^ | Ixazomib-Rd | 1.5 | 138 | 68 | NR | NR | NR | 7.2 | 27.6 | 27.6 | NR |
| Canadian provincial MM program^56^ | Any novel-agent based regimen | 1 | 355 | NR | NR | NR | NR | NR | 12 | NR | 36 |
|  |  | 2 | 213 | NR | NR | NR | NR | NR | 8 | NR | 20 |
|  |  | 3 | 127 | NR | NR | NR | NR | NR | 6 | NR | 12 |
| US national database analysis^57^ | Rd | NR | 283 | NR | NR | NR | NR | NR | NR | NR | 43.9 |
|  | Vd | NR | 247 | NR | NR | NR | NR | NR | NR | NR | 30.8 |
| US Oncology database analysis^58^ | KRd | 1 | 112 | 64.7 | 23/36/33 | NR | 10 | NR | 25.3 | NR | NR |
|  | VRd | 1 | 27 | 62.4 | 26/26/33 | NR | 15 | NR | 10.2 | NR | NR |
|  | VCd | 1 | 17 | 69.1 | 18/29/29 | NR | 6 | NR | 6.5 | NR | NR |
| US national database analysis^59^ | Rd | 1.9 | 773 | 69 | NR | NR | NR | 10.1 | 11.6/14.3 | NR | 35.4 |
|  | Vd | 2 | 694 | 69 | NR | NR | NR | 6.3 | 7.7/10 | NR | 24.9 |
| Turkish multi-center retrospective review^60^ | Pom-based | 5 | 117 | 68 | 21/47/33 | 29 | NR | >2 cycles (NR) | 5.6 | NR | 8.4 |
| Turkish multi-center retrospective review^61^ | Cfz | 4 | 152 | 69 | 22/51/26 | 18 | NR | >2 cycles (NR) | 6 | NR | 10.4 |
| US EMR database analysis^62^ | VRd | 1 | 343 | 69 | 30//7 | 12 | 44 | 8.7 | 13.6 | NR | NR |
|  | KRd | 1 | 139 | 65 | 19//4 | 28 | 39 | 7.2 | 10.2 | NR | NR |
|  | Ixazomib-Rd | 2 | 49 | 73 | 10//6 | 22 | 37 | NE | NE | NR | NR |
| EHA 2017 |  |  |  |  |  |  |  |  |  |  |  |
| Greek Myeloma Study Group analysis^36^ | Ixazomib-Rd | 1* | 41 | 70.5 | NR | NR | NR | 7.12 | 90.5% at 6 months | NR | NR |
| Polish Myeloma Group compassionate use program^37^ | Dara | 4* | 30 | NR | NR | NR | NR | 4.4 | NR | NR | NR |
| Observational study^38^ | Btz-based | ≥1 | 326 | NR | NR | NR | NR | 4.5 | 7 | NR | NR |
|  | Cfz-based |  | 86 | NR | NR | NR | NR | 3.4 | 5.6 | NR | NR |
| Czech Myeloma Group retrospective analysis^39^ | Pom-dex | 4* | 122 | 67 | NR | NR | NR | NR | NR | 7.1 | 19 |
| Dutch population-based registry^40^ | Pom-dex | ≥2 | 82 | NR | NR | NR | 9.8 | 3 cycles (28 days) | 3.8 | NR | NR |
| Turkish retrospective single-center study^41^ | Rd | 1* | 120 | 64 | NR | NR | NR | NR | 21 | NR | 32 |
| UK retrospective single-center analysis^42^ | Ixazomib-Rd | 2* | 30 | 65 | 60 / 13 / 27 | 69 | NR | 6 cycles | 19.2 | NR | NR |
| Italian real-life experience^43^ | Cfz-Rd | 3* | 21 | 62 | NR | NR | NR | NR | NR | NR | 3 |
| ASCO 2017 |  |  |  |  |  |  |  |  |  |  |  |
| EMR review^14^ | Pano-Vd | 6* | 37 | 62 | NR | 27 | NR | 2 cycles (21 days) | 2.4 | NR | 7.5 |
| Single-center retrospective chart review^16^ | Ven-Btz | 9* | 11 | NR | NR | 9 | NR | 14.24 wks | NR | NR | NR |
| Single-center retrospective review^15^ | Dara-based | 4* | 130 | 67 | NR | NR | NR | NR | 5.5/5.9 | NR | NR |
| IMW 2017 |  |  |  |  |  |  |  |  |  |  |  |
| Italian retrospective real-life analysis^29^ | Bendamustine-Vd | 6* | 56 | 57 | NR | NR | NR | NR | NR | NR | 9.8 |
| US EMR database analysis^30^ | Len-based (no PI) | 1 | 227 | 69 | NR | NR | NR | 10.1 | 20.1 | NR | NR |
|  | Btz-based (+/-len) | 1 | 213 | 72 | NR | NR | NR | 6.6 | 11.9 | NR | NR |
|  | Cfz-based (+/-len) | 1 | 52 | 67 | NR | NR | NR | 4.6 | 5.7 | NR | NR |
| Single-center retrospective review^31^ | Pano-Vd | 5* | 24 | 67 | 45 / 55 (II+III) | 58 | NR | NR | 3.3 | NR | 9.8 |
| Retrospective medical records analysis^32^ | Rd | 1 | 135 | 69 | 27 / 36 / 36 | 16 | NR | NR | 19.2 | NR | NR |
| ASH 2016 |  |  |  |  |  |  |  |  |  |  |  |
| Single-center retrospective review^19^ | Cfz-Pom-dex | 4* | 45 | 65 | NR | NR | NR | 2 | 3.3/3.5 | NR | 16.1 |
| Czech Myeloma Group chart data^20^ | All | 4* | 463 | 62 | NR | NR | NR | NR | 5.6 | NR | 20.1 |
|  | Dara-based | 5* | 148 | 64 | NR | NR | NR | NR | 4.0 | NR | 11.9 |
| Observational study^21^ | 57% PI, 26% IMID, 17% both | 2* | 239 | 66 | 16 / 15 / 27 | NR | NR | NR | 7.9 | NR | 22.3% at 18 months |
| Observational study^22^ | PI/IMID/both | 1 | 367 | 70 | NR | NR | NR | NR | 11.5/14.1 | NR | NR |
| Retrospective observational study^23^ | Btz-based retreat | 1 | 135 | 61 | 30 / 30 / 40 | NR | 14 | 6 cycles  (NR) | 19 | NR | NR |
| Real-life retrospective evaluation^24^ | Dara | 4 | 25 | 67 | NR | NR | NR | NR | NR | NR | NR |
|  | Dara-Pom-dex | 4 | 39 | 71 | NR | NR | NR | NR | NR | NR | NR |
| EHA 2016 |  |  |  |  |  |  |  |  |  |  |  |
| Observational study^44^ | PI/IMiD/both | 1* | 764 | 68 | NR / NR / 40 | NR | NR | NR | 9.3 | NR | NR |
| Czech Myeloma Group retrospective registry analysis^45^ | All | 1 / 2 | 1118 | NR | NR | NR | NR | NR | NR | NR | NR / 13.2 |
|  | Btz-based | 1 | NR | NR | NR | NR | NR | NR | 11.3 | NR | 27.3 |
|  |  | 2 | NR | NR | NR | NR | NR | NR | 5.7 | NR | 16.2 |
|  | Len-based | 1 | NR | NR | NR | NR | NR | NR | 8.7 | NR | 26.2 |
|  |  | 2 | NR | NR | NR | NR | NR | NR | 6.6 | NR | 12.6 |
| US retrospective EMR cohort study^46^ | PI/IMID/both | 1 | 628 | 69.2 | NR | 9.6 | NR | 6.9 | 15.1 | NR | 41 |
|  |  | 2 | NR | NR | NR | NR | NR | 5.5 | 7.8 | NR | 30.3 |
|  |  | 3 | NR | NR | NR | NR | NR | NR | 6.9 | NR | 24.8 |
| Dutch population-based registry^47^ | All | 1 | 1522 | 71 | NR | NR | NR | NR | 8.9 | NR | 19.7 |
|  |  | 2 | NR | 71 | NR | NR | NR | NR | 6.4 | NR | 13.9 |
|  |  | 3 | NR | 72 | NR | NR | NR | NR | NR | NR | NR |
| US retrospective EMR cohort study^48^ | All | 1 | 628 | NR | NR | NR | NR | 6.9 | NR | NR | NR |
|  |  | 2 | NR | NR | NR | NR | NR | 5.5 | NR | NR | NR |
| Prospective non-interventional study^49^ | Pom-dex (prior len) | 2* | 126 total | 74 | NR | NR | NR | NR | NR | NR | NR |
|  | Pom-dex (no prior len) | 4* | 126 total | 74 | NR | NR | NR | NR | NR | NR | NR |
| ASCO 2016 |  |  |  |  |  |  |  |  |  |  |  |
| Retrospective cohort study, Medicare database^17^ | All (48% PI and/or IMID) | 1 or 2 | 4985 | NR | NR | NR | NR | NR | NR | NR | 23.9 / 15.5 |
| Retrospective cohort study, US national EMR database^18^ | 46% IMID, 35% PI, 6% both | 1 | 340 | 70 | NR | 9 | NR | 6.87 | NR | NR | 61% to 90% at 1 year |
| ASH 2015 |  |  |  |  |  |  |  |  |  |  |  |
| Canadian real-world cohort^25^ | All, prior ASCT | 1 | 127 total | NR | NR | NR | NR | NR | 12 | NR | 35 |
|  | All, no prior ASCT | 1 | 127 total | NR | NR | NR | NR | NR | 11 | NR | 25 |
|  | All, prior ASCT | 2 | 64 total | NR | NR | NR | NR | NR | 6 | NR | 17 |
|  | All, no prior ASCT | 2 | 64 total | NR | NR | NR | NR | NR | 6 | NR | 15 |
| French retrospective single-center experience^26^ | Pom-cyclo-dex | 4* | 20 | 57 | 50 / 30 / 20 | NR | 10 | 4 | 81% at 1 year | NR | NR |
| IMWG global multicenter retrospective study^27^ | All | 1 | 307 | 59 | NR | NR | NR | NR | 13.4 / 15 | NR | 36.1 |
|  |  | 2 | 163 | NR | NR | NR | NR | NR | 8.3 / 9.8 | NR | 18.4 |
|  |  | 3 | 84 | NR | NR | NR | NR | NR | 6.4 / 6.6 | NR | 12.6 |
| Retrospective cohort study, US national claims database^28^ | PI/IMID-based | 1 | 249 | NR | NR | NR | NR | 6.3 | NR | NR | 82% at 1 year |
| IMW 2015 |  |  |  |  |  |  |  |  |  |  |  |
| German single-center access program^33^ | Cfz | 6* | 22 | NR | NR | NR | NR | 4 cycles (28 days) | 6.0 | NR | 14.9 |
| Analysis from 4 UK regional hospitals^34^ | Pom-dex | 3* | 38 | 69 | NR | NR | NR | 4 cycles (28 days) | 3.4 | NR | 10.9 |
| Analysis from 2 UK tertiary hematology centers^35^ | Rd | 3* | 140 | 63 | NR | NR | NR | 7 cycles (28 days) | 11.7 | NR | 25.7 |

*Median, except where stated.

ASCT, autologous stem cell transplantation; Btz, bortezomib; Cfz, carfilzomib; cyclo, cyclophosphamide; cyto, cytogenetics; Dara, daratumumab; dex, dexamethasone; DOT, duration of therapy; EMR, electronic medical record; HR, high-risk; IMiD, immunomodulatory drug; ISS, International Staging System; Len, lenalidomide; LOT, lines of therapy; NR, not reported; OS, overall survival; Pano, panobinostat; PFS, progression-free survival; PI, proteasome inhibitor; Pom, pomalidomide; Rd, lenalidomide, dexamethasone; RI, renal impairment or renal failure; Thal, thalidomide; TTNT, time to next therapy; TTP, time to progression; Vd, bortezomib, dexamethasone; Ven, venetoclax; wks, weeks.

**Supplementary Table S3:** Publications reporting phase 3 clinical trial data on treatment duration and/or outcomes in patients with RRMM, with full study citations

| Study | Regimen | Prior lines* | N | Age* | ISS I / II / III, % | HR cyto, % | RI, % | DOT* | PFS,* mos | TTP,* mos | OS,* mos |
| --- | --- | --- | --- | --- | --- | --- | --- | --- | --- | --- | --- |
| APEX^63,64^ | Btz | 2 | 333 | 62 | NR | NR | NR | ~15 weeks | NR | 6.2 | 29.8 |
|  | Dex | 2 | 336 | 61 | NR | NR | NR | ~15 weeks | NR | 3.5 | 23.7 |
| MMY-3021^65,66^ | SC btz ± dex | 1 | 148 | 64.5 | 27 / 41 / 32 | 14 | 41 | ~24 weeks | 9.3 | 9.7 | 28.7 |
|  | IV btz ± dex | 1 | 74 | 64.5 | 27 / 41 / 32 | 19 | 32 | ~24 weeks | 8.4 | 9.6 | NE |
| ASPIRE^67,68^ | Cfz-Rd | 2 | 396 | 64 | 16.2 / 25.0 / 46.7 | 12.1 | 6.3 | 88 weeks | 26.3 | 31.4 | 48.3 |
|  | Rd | 2 | 396 | 65 | 18.7 / 23.7 / 40.7 | 13.1 | 8.1 | 57 weeks | 17.6 | 19.4 | 40.4 |
| ENDEAVOR^67,69^ | Cfz-dex | 2 | 464 | 65 | 44 / 56 (II+III) | 21 | 18 | 39.9 weeks | 18.7 | NR | 47.6 |
|  | Vd | 2 | 465 | 65 | 44 / 56 (II+III) | 24 | 21 | 26.8 weeks | 9.4 | NR | 40.0 |
| FOCUS^70^ | Cfz | 5 | 157 | 63 | 17 / 20 / 42 | 14 | 11 | 16.3 weeks | 3.7 | NR | 10.2 |
|  | BSC | 5 | 158 | 66 | 13 / 26 / 35 | 18 | 9 | 10.7 weeks | 3.3 | NR | 10.0 |
| TOURMALINE-MM1^71^ | Ixazomib-Rd | 1 | 360 | 66 | 63 / 25 / 12 | 21 | 22 | ~68 weeks | 20.6 | 21.4 | NR |
|  | Placebo-Rd | 1 | 362 | 66 | 64 / 24 / 12 | 17 | 27 | ~60 weeks | 14.7 | 15.7 | NR |
| ELOQUENT-2^72,73^ | Elo-Rd | 2 | 321 | 67 | 44 / 32 / 21 | NR | 30 | 17 months | 19.4 | NR | 48.0 |
|  | Rd | 2 | 325 | 66 | 42 / 32 / 21 | NR | 23 | 12 months | 14.9 | NR | 40.0 |
| CASTOR^74^ | Dara-Vd | 2 | 251 | 64 | 39.0 / 37.5 / 23.5 | 22.7 | 30 | NR | NE | NE | NE |
|  | Vd | 2 | 247 | 64 | 38.9 / 40.5 / 20.6 | 21.3 | 23 | NR | 7.2 | 7.3 | NE |
| POLLUX^75^ | Dara-Rd | 1 | 286 | 65 | 47.9 / 32.5 / 19.6 | 15.4 | NR | NR | NE | NR | NE |
|  | Rd | 1 | 283 | 65 | 49.5 / 30.4 / 20.1 | 16.6 | NR | NR | 18.4 | NR | NE |
| PANORAMA-1^76,77^ | Pano-Vd | 1 | 387 | 63 | 40 / 27 / 20 | 18 | None |  | 12.0 | 12.7 | 40.3 |
|  | Placebo-Vd | 1 | 381 | 63 | 40 / 24 / 23 | 18 | None |  | 8.1 | 8.5 | 35.8 |
| MM-009/MM-010^78-80^ | Rd | 62% ≥2 / 2 | 177/176 | 64/63 | NR | NR | NR | NR | NR | 11.1/11.3 | 38.0 |
|  | Dex | 62% ≥2 / 2 | 176/175 | 62/64 | NR | NR | NR | NR | NR | 4.7/4.7 | 31.6 |
| MM-003 (NIMBUS)^81^ | Pom-dex | 5 | 302 | 64 | 65 (I+II) / 31 | NR | 31 | NR | 4.0 | 4.7 | 12.7 |
|  | Dex | 5 | 153 | 65 | 61 (I+II) / 35 | NR | 39 | NR | 1.9 | 2.1 | 8.1 |
| STRATUS^82^ | Pom-dex | 5 | 682 | 66 | 60.7 (I+II) / 34.6 | NR | 34.8 | 4.9 months | 4.6 | NR | 11.9 |

*Median, except where stated.

BSC, best supportive care; Btz, bortezomib; Cfz, carfilzomib; cyto, cytogenetics; Dara, daratumumab; dex, dexamethasone; DOT, duration of therapy; HR, high-risk;ISS, International Staging System; NE, not estimable; NR, not reported; OS, overall survival; Pano, panobinostat; PFS, progression-free survival; Pom, pomalidomide; Rd, lenalidomide, dexamethasone; RI, renal impairment or renal failure; TTP, time to progression; Vd, bortezomib, dexamethasone.

**Supplementary Table S4:** Comparison of PFS/TTNT from real-world reports and phase 3 clinical studies in RRMM patients after 1–3 prior lines of therapy, with full study citations

| Regimens | Phase 3 clinical studies | Real-world reports | | |
| --- | --- | --- | --- | --- |
|  |  | **All reports identified** | **Studies/registry analyses*** | **EMR/chart review/claims analyses†** |
| All regimens combined | Not applicable | 6–15.1 | 6.4–14.1 | 6–15.1 |
| PI doublet / PI-based‡ | Btz: 6.2–9.4  Cfz: 14.9–22.2 | Btz: 5.7–11.9  Cfz: 3.2–10.6 | Btz: 5.7–11.3  Cfz: 5.6–10.6 | Btz: 6.9–11.9  Cfz: 3.2–9.4 |
| PI-alkylator triplet | 12–18.4^#^ | 6.5–16.2 | NR | 6.5–16.2 |
| Injectable PI-immunomodulatory drug triplet | 18.3–29.6 | 9.4–25.3 | NR | 9.4–25.3 |
| Oral PI-immunomodulatory drug triplet | 17.5–20.6 | 19.2–27.6 | 27.6 | 19.2 |
| Len doublet / len-based‡ | 11.1–18.4 | 6.6–21 | 6.6–8.7 | 7–21 |

Data shown are ranges of median PFS/TTNT (months) reported from multiple studies/analyses.

*Including prospective and retrospective registry studies and observational studies, and analyses of data from named patient programs/compassionate use programs

†Including single-center, retrospective chart reviews, EMR reviews, longitudinal chart reviews

‡Regimen not specified beyond ‘PI-based’ or ‘len-based’ in some real-world reports. ^#^Data from two phase 2 studies of VCd.

For ease of reading, references cited are shown here:

All regimens combined: All reports identified;^1,2,21,22,25,27,44,47,48,56^ Studies/registry analyses; ^21,22,44,47^ EMR/chart review/claims analyses^1,2,25,27,48,56^

PI doublet/PI-based (Btz): Phase 3 clinical studies;^64,65,69,74,76^ All reports identified;^1,30,38,45,59^ Studies/registry analyses;^38,45^ EMR/chart review/claims analyses^1,30,59^

PI doublet/PI-based (Cfz): Phase 3 clinical studies;^83^ All reports identified;^1,5,30,38,51,53^ Studies/registry analyses;^38,51^ EMR/chart review/claims analyses^1,5,30,53^

PI-alkylator triplet: Phase 3 clinical studies;^84,85^ All reports identified;^8,58^ EMR/chart review/claims analyses^8,58^

Injectable PI-immunomodulatory drug triplet: Phase 3 clinical studies;^86,87^ All reports identified;^1,58,62^ EMR/chart review/claims analyses^1,58,62^

Oral PI-immunomodulatory drug triplet: Phase 3 clinical studies;^71^ All reports identified;^42,55^ Studies/registry analyses;^55^ EMR/chart review/claims analyses^42^

Len doublet/len-based: Phase 3 clinical studies;^68,71,73,74,79^ All reports identified;^1,3,30,32,35,41,45,59^ Studies/registry analyses;^45^ EMR/chart review/claims analyses^1,3,30,32,35,41,59^

**Supplementary Table S5:** Comparison of DOT from real-world reports and phase 3 clinical studies in RRMM patients after 1–3 prior lines of therapy, with full study citations

| Regimens | Phase 3 clinical studies | Real-world reports | | |
| --- | --- | --- | --- | --- |
|  |  | **All reports identified** | **Studies/registry analyses*** | **EMR/chart review analyses†** |
| All regimens combined | Not applicable | 2.1–7 | 4.6 | 2.1–7 |
| PI doublet / PI-based‡ | Btz: 4–6.2  Cfz: 9.2 | Btz: 3.7–6.6  Cfz: 2.3–4.6 | Btz: 4.1–4.5  Cfz: 3.4 | Btz: 3.7–6.6  Cfz: 2.3–4.6 |
| PI-alkylator triplet | NR^#^ | 5 | NR | 5 |
| Injectable PI-immunomodulatory drug triplet | 5.2–20.3 | 3.4–8.7 | 5 | 3.4–8.7 |
| Oral PI-immunomodulatory drug triplet | 15.7 | 5.5–7.2 | 5.5^§^–7.2 | 5.5 |
| Len doublet / len-based‡ | 10.1–13.8 | 3.1–16.8 | 4.9–5.6 | 3.1–16.8 |

Data shown are ranges of median DOT (in months) reported from multiple studies/analyses.

*Including prospective and retrospective registry studies and observational studies, and analyses of data from named patient programs/compassionate use programs

†Including single-center, retrospective chart reviews, EMR reviews, longitudinal chart reviews

‡Regimen not specified beyond ‘PI-based’ or ‘len-based’ or ‘IMiD-based’ in some real-world reports. ^#^Data from two phase 2 studies of VCd. §Cycles (28-day).

For ease of reading, references cited are shown here:

All regimens combined: All reports identified;^1,2,46,48,50^ Studies/registry analyses;^50^ EMR/chart review/claims analyses^1,2,46,48^

PI doublet/PI-based (Btz): Phase 3 clinical studies;^64,65,69,74,76^ All reports identified;^1,30,38,50,57^ Studies/registry analyses;^38,50^ EMR/chart review/claims analyses^1,30,57^

PI doublet/PI-based (Cfz): Phase 3 clinical studies;^69^ All reports identified;^1,5,30,38,53^ Studies/registry analyses;^38^ EMR/chart review/claims analyses^1,5,30,53^

PI-alkylator triplet: Phase 3 clinical studies;^84,85^ All reports identified;^8^ EMR/chart review/claims analyses^8^

Injectable PI-immunomodulatory drug triplet: Phase 3 clinical studies;^86,87^ All reports identified;^1,28,50,62^ Studies/registry analyses;^50^ EMR/chart review/claims analyses^1,28,62^

Oral PI-immunomodulatory drug triplet: Phase 3 clinical studies;^71^ All reports identified;^42,54,55^ Studies/registry analyses;^54,55^ EMR/chart review/claims analyses^42^

Len doublet/len-based: Phase 3 clinical studies;^68,71,73,74,79^ All reports identified;^1,3,4,30,32,35,50,57^Studies/registry analyses;^4,50^ EMR/chart review/claims analyses^1,3,30,32,35,57^

**References**

1. Jagannath S, Roy A, Kish J, Lunacsek O, Globe D, Eaddy M*, et al.* Real-world treatment patterns and associated progression-free survival in relapsed/refractory multiple myeloma among US community oncology practices. *Expert Rev Hematol* 2016 Jul; **9**(7)**:** 707-717.

2. Yong K, Delforge M, Driessen C, Fink L, Flinois A, Gonzalez-McQuire S*, et al.* Multiple myeloma: patient outcomes in real-world practice. *Br J Haematol* 2016 Oct; **175**(2)**:** 252-264.

3. Mele G, Melpignano A, Quarta G, Palumbo G, Capalbo S, Falcone A*, et al.* "Real world" outcome of lenalidomide plus dexamethasone in the setting of recurrent and refractory multiple myeloma: extended follow-up of a retrospective multicenter study by the "rete ematologica pugliese". *Leuk Res* 2015 Mar; **39**(3)**:** 279-283.

4. Alegre A, Aguado B, Giraldo P, Rios E, Canovas A, Ibanez A*, et al.* Lenalidomide is effective as salvage therapy in refractory or relapsed multiple myeloma: analysis of the Spanish Compassionate Use Registry in advanced patients. *Int J Hematol* 2011 Mar; **93**(3)**:** 351-360.

5. Chen CC, Parikh K, Abouzaid S, Purnomo L, McGuiness CB, Hussein M*, et al.* Real-World Treatment Patterns, Time to Next Treatment, and Economic Outcomes in Relapsed or Refractory Multiple Myeloma Patients Treated with Pomalidomide or Carfilzomib. *J Manag Care Spec Pharm* 2017 Feb; **23**(2)**:** 236-246.

6. Katodritou E, Vadikolia C, Lalagianni C, Kotsopoulou M, Papageorgiou G, Kyrtsonis MC*, et al.* "Real-world" data on the efficacy and safety of lenalidomide and dexamethasone in patients with relapsed/refractory multiple myeloma who were treated according to the standard clinical practice: a study of the Greek Myeloma Study Group. *Ann Hematol* 2014 Jan; **93**(1)**:** 129-139.

7. Maciocia N, Melville A, Cheesman S, Sharpley F, Ramasamy K, Streetly M*, et al.* Real-world use of pomalidomide and dexamethasone in double refractory multiple myeloma suggests benefit in renal impairment and adverse genetics: a multi-centre UK experience. *Br J Haematol* 2017 Mar; **176**(6)**:** 908-917.

8. Reece DE, Trieu Y, Masih-Khan E, Atenafu EG, Chen C, Prica A*, et al.* Cyclophosphamide and Bortezomib With Prednisone or Dexamethasone for the Treatment of Relapsed and Refractory Multiple Myeloma. *Clin Lymphoma Myeloma Leuk* 2016 Jul; **16**(7)**:** 387-394.

9. Reyal Y, Popat R, Cheesman S, Rismani A, D'Sa S, Rabin N*, et al.* Real world experience of bortezomib re-treatment for patients with multiple myeloma at first relapse. *Br J Haematol* 2017 May; **177**(3)**:** 495-497.

10. Usmani S, Ahmadi T, Ng Y, Lam A, Desai A, Potluri R*, et al.* Analysis of Real-World Data on Overall Survival in Multiple Myeloma Patients With >/=3 Prior Lines of Therapy Including a Proteasome Inhibitor (PI) and an Immunomodulatory Drug (IMiD), or Double Refractory to a PI and an IMiD. *Oncologist* 2016 Aug 02.

11. Scott A, Weber N, Tiley C, Taylor K, Taper J, Harrison S*, et al.* 'Real-world' Australian experience of pomalidomide for relapsed and refractory myeloma. *Leuk Lymphoma* 2018 Oct 12; **59**(6)**:** 1514-1516.

12. Blimark CH, Turesson I, Genell A, Ahlberg L, Bjorkstrand B, Carlson K*, et al.* Outcome and survival of myeloma patients diagnosed 2008-2015. Real-world data on 4904 patients from the Swedish Myeloma Registry. *Haematologica* 2018 Mar; **103**(3)**:** 506-513.

13. Matsumura-Kimoto Y, Kuroda J, Kaneko H, Kamitsuji Y, Fuchida SI, Nakaya A*, et al.* Pomalidomide with or without dexamethasone for relapsed/refractory multiple myeloma in Japan: a retrospective analysis by the Kansai Myeloma Forum. *Int J Hematol* 2018 Jan 29; **107**(5)**:** 541-550.

14. Biran N, Vesole DH, Zhang S, Richter JR, Kuo YH, Schmidt L*, et al.* Real-world outcomes with panobinostat in patients with penta- and quad-refractory multiple myeloma. *J Clin Oncol* 2017; **35**(suppl)**:** e19522.

15. Lakshman A, Abeykoon JP, Kumar S, Rajkumar SV, Kourelis T, Buadi F*, et al.* Daratumumab-based combination therapies (DCT) in heavily-pretreated patients (pts) with relapsed and/or refractory multiple myeloma (RRMM). *J Clin Oncol* 2017; **35**(suppl)**:** 8038.

16. Galligan D, Shah N, Wolf JL, Wong SWK, Abramovitz L, Donnelly B*, et al.* A single-center retrospective cohort analysis of venetoclax in post-transplant, relapsed/refractory multiple myeloma. *J Clin Oncol* 2017; **35**(suppl e19514).

17. Lin J, Lingohr-Smith M, Omar M, Chen C. Survival of U.S. Medicare patients with multiple myeloma by line of therapy. *J Clin Oncol* 2016; **34**(suppl e18098).

18. Hari P, Tran LM, Yong C, Noga SJ, Farrelly E, Raju A*, et al.* Duration of second line treatment and survival in multiple myeloma. *J Clin Oncol* 2016; **34**(suppl)**:** e18107.

19. Hobbs M, Paludo J, Fonder A, Abeykoon JP, Gertz MA, Rajkumar SV*, et al.* Efficacy of Carfilzomib (K), Pomalidomide (P), and Dexamethasone (d) in Heavily Pretreated Patients with Relapsed/ Refractory Multiple Myeloma (RRMM) in a Real World Setting. *Blood* 2016; **128**(22)**:** 3337.

20. Hájek R, Jelinek T, Maisnar V, Pour L, Spicka I, Minarik J*, et al.* Comparative Effectiveness of Daratumumab Monotherapy Versus a Real-World Historical Control from the Czech Republic in Heavily Pretreated and Highly Refractory Multiple Myeloma Patients. *Blood* 2016; **128**(22)**:** 3332.

21. Vij R, Khare S, Perez S, Popov S, Chen C. Subgroup Analysis of US Patients in Preamble, an Ongoing Multinational Observational Study in Multiple Myeloma. *Blood* 2016; **128**(22)**:** 2362.

22. Durie B, Kuter DJ, Davis C, Zyczynski T, Goldschmidt J, Vij R*, et al.* An Ongoing, Observational Cohort Study in Multiple Myeloma (PREAMBLE): Preliminary Efficacy Analyses in Patients with 1 Line of Prior Therapy. *Blood* 2016; **128**(22)**:** 2403.

23. Musto P, Simeon V, Cascavilla N, Falcone A, Petrucci MT, Cesini L*, et al.* Real Life, Retrospective Analysis of Bortezomib Re-Use As Second Treatment for Relapsed Multiple Myeloma Patients Previously Exposed to Bortezomib-Based Therapies As First Line: The Rebound Study. *Blood* 2016; **128**(22)**:** 4494.

24. Branca A, Buros A, Yoon D, Suva LJ, Weinhold N, Rasche L*, et al.* Daratumumab Single Agent and Daratumumab Plus Pomalidomide and Dexametasone in Relapsed/Refractory Multiple Myeloma: A Real Life Retrospective Evaluation. *Blood* 2016; **128**(22)**:** 4516.

25. Venner CP, Bahlis NJ, Neri P, Sandhu I, Duggan P, Belch A*, et al.* In Multiple Myeloma Progression Free and Overall Survival in the Relapsed Setting Remains Poor with Early Exposure to Novel Agents: Experience from a Real-World Cohort. *Blood* 2015; **126**(23)**:** 4261.

26. Garderet L, Polge E, Gueye mS, Kellil C, Ova JGO, Beohou E*, et al.* Pomalidomide, Cyclophosphamide and Dexamethasone for Relapsed/Refractory Multiple Myeloma: A Retrospective Single Center Experience. *Blood* 2015; **126**(23)**:** 1858.

27. Kumar SK, Lee JH, Dimopoulos MA, Terpos E, Kastritis E, Chng WJ*, et al.* Outcomes after Initial Relapse of Multiple Myeloma: An International Myeloma Working Group Study. *Blood* 2015; **126**(23)**:** 4201.

28. Romanus D, Jhaveri M, Labotka R, Henk H, Seal B. Treatment Patterns and Outcomes Among Patients Who Initiate Therapy for Relapsed/Refractory Multiple Myeloma (RRMM) in Routine Care. *Blood* 2015; **126**(23)**:** 4515.

29. Cerchione C, Catalano L, Pareto AE, Basile S, Marano L, Peluso I*, et al.* Bendamustine-Bortezomib-Dexamethasone (BVD) in the Management of Relapsed and Refractory Multiple Myeloma: A Real-Life Experience. *Clin Lymphoma Myeloma Leuk* 2017; **17**(1)**:** e60-e61.

30. Romanus D, Raju A, Yong C, Farrelly E, Luptakova K, Labotka R*, et al.* Association Between Treatment Regimen Type in Second-Line Therapy (2LT) and Duration of Therapy (DOT) & Time To Next Treatment (TTNT) in a United States (US) Relapsed/Refractory Multiple Myeloma (RRMM) Cohort. *Clin Lymphoma Myeloma Leuk* 2017; **17**(1)**:** e81-e82.

31. Baertsch MA, Hillengass J, Schoenland S, Hegenbart U, Ho AD, Goldschmidt H*, et al.* Efficacy and Tolerability of the Histone-Deacetylase Inhibitor Panobinostat in Clinical Practice. *Clin Lymphoma Myeloma Leuk* 2017; **17**(1)**:** e119-e120.

32. Katodritou E, Delimpasi S, Kyrtsonis MC, Kyriakou D, Symeonidis A, Spanoudakis E*, et al.* Real-World data for the Treatment of Relapsed/Refractory Multiple Myeloma with Lenalidomide and Dexamethasone in 2nd Line (Legend Study): The Prognostic Significance of Biochemical Versus Clinical Relapse. *Clin Lymphoma Myeloma Leuk* 2017; **17**(1)**:** e130-e131.

33. Danhof S, Schreder M, Rasche L, Strifler S, Einsele H, Knop S. Efficacy and Safety of Pre-approval Carfilzomib-Based Therapy of Multiple Myeloma in Daily Practice - Experience from a German Academic Center. *Clin Lymphoma Myeloma Leuk* 2015; **15**(Suppl 3)**:** e284.

34. Miles O, Wells M. Efficacy of Pomalidomide after Progression Following Lenalidomide and Bortezomib-a Multicenter Retrospective Study. *Clin Lymphoma Myeloma Leuk* 2015; **15**(Suppl 3)**:** e302.

35. Chadwick JA, Krishna R, Tholouli E, Kulkarni S, Thornton E, Sinacola A*, et al.* Lenalidomide for heavily pretreated relapsed/refractory myeloma cohort in northwest United Kingdom (UK) - efficacy & tolerability: high response & low second malignancy rates. *Clin Lymphoma Myeloma Leuk* 2015; **15**(Suppl 3)**:** e302-e303.

36. Terpos, E., Katodritou E, Kotsopoulou M, Ntanasis-Stathopoulos I, Lampropoulou P, Papadaki S*, et al.* "Real world" data on the efficacy and safety of ixazomib in combination with lenalidomide and dexamethasone in relapsed/refractory multiple myeloma: a study of the Greek Myeloma Study Group. *Haematologica* 2017; **102**(s2)**:** 511-512.

37. Salomon-Perzyński A, Walter-Croneck A, Usnarska-Zubkiewicz L, Dytfeld D, Zielińska P, Wojciechowska M*, et al.* Real-world results of daratumumab monotherapy in heavily pretreated relapsed/refractory multiple myeloma in Poland: a prospective observational study of the Polish Myeloma Group. *Haematologica* 2017; **102**(s2)**:** 517.

38. Durie B, Cook G, Goldschmidt H, Kuter D, Zyczynski T, Popov S*, et al.* The use of carfilzomib and bortezomib in routine clinical practice: results from PREAMBLE, an ongoing, observational cohort study in multiple myeloma. *Haematologica* 2017; **102**(s2)**:** 520.

39. Pour L, Brozova L, Spicka I, Maisnar V, Minarik J, Jungova A*, et al.* Pomalidomid is more effective in real clinical practice than in randomized trial - an observational study of the Czech Myeloma Group. *Haematologica* 2017; **102**(s2)**:** 529.

40. Wester R, Dinmohamed A, Sonneveld P, Broijl A, Blijlevens N. Pomalidomide with low-dose dexamethasone in patients with relapsed or relapsed and refractory multiple myeloma: a prospective analysis in a population-based registry. *Haematologica* 2017; **102**(s2)**:** 533-534.

41. Saydam G, Soyer N, Patır P, Uysal A, Duran M, Durusoy R*, et al.* Efficacy and safety of lenalidomide and dexamethasone in patients with relapsed/refractory multiple myeloma: a real life experience from Turkey. *Haematologica* 2017; **102**(s2)**:** 784.

42. Ziff M, Cheesman S, Kyriakou C, Mehta A, Papanikolaou X, Rabin N*, et al.* Real world use of ixazomib with lenalidomide and dexamethasone for patients with relapsed and relapsed refractory multiple myeloma. *Haematologica* 2017; **102**(s2)**:** 786-787.

43. Cerchione C, Ferrara K, Peluso I, Nappi D, Di Perna M, Zacheo I*, et al.* Carfilzomib-lenalidomide-dexamethasone in the management of relapsed and refractory multiple myeloma: a real-life experience. *Haematologica* 2017; **102**(s2)**:** 804.

44. Kuter D, Cook G, Goldschmidt H, Cella D, Zyczynski T, Davis C*, et al.* An ongoing multinational observational study in multiple myeloma (PREAMBLE): preliminary report on progression-free survival. *Haematologica* 2016; **101**(s1)**:** 520.

45. Hajek R, Jarkovsky J, Maisnar V, Pour L, Spicka I, Minařík J*, et al.* Survival and treatment patterns in patients with symptomatic multiple myeloma (MM) in a real-world setting. *Haematologica* 2016; **101**(s1)**:** 529.

46. Romanus D, Raju A, Seal B, Farrelly E, Yong C, Noga S*, et al.* The clinical course of relapsed or refractory US multiple myeloma (RRMM) patients receiving two or more lines of therapy. *Haematologica* 2016; **101**(s1)**:** 532.

47. Verelst S, Blommestein H, Gonzalez-McQuire S, DeCosta L, de Raad J, Sonneveld P*, et al.* Overall survival in patients with symptomatic multiple myeloma in the real-world setting: a retrospective analysis of the PHAROS registry in the Netherlands. *Haematologica* 2016; **101**(s1)**:** 534-535.

48. Romanus D, Raju A, Yong C, Seal B, Farrelly E, Noga S*, et al.* Duration of therapy in U.S. patients treated for relapsed/refractory multiple myeloma (RRMM) in the real-world. *Haematologica* 2016; **101**(s1)**:** 538-539.

49. Dechow T, Aldaoud A, Hurtz HJ, Knauf W, Groschek M, Hansen R*, et al.* Overall response rate of patients with refractory multiple myeloma treated with pomalidomide and low dose dexamethasone after lenalidomide failure: interim results of the POSEIDON study. *Haematologica* 2016; **101**(s1)**:** 540.

50. Kuter DJ, Chen C, Popov S, Davis C, Vij R. Healthcare Resource Utilization and Costs Associated with Different Treatment Modalities of Relapsed/Refractory Multiple Myeloma Patients in the US: Findings from Preamble. *Blood* 2017; **130**(suppl)**:** 3157.

51. Rifkin RM, Amirian ES, Medhekar R, Wilson T, Boyd M, Mezzi K*, et al.* Carfilzomib Dosing Patterns and Time to Next Treatment Among Adult Patients with Multiple Myeloma Treated in the US Community Oncology Setting. *Blood* 2017; **130**(suppl)**:** 3433.

52. Minarik J, Pour L, Maisnar V, Spicka I, Jungova A, Plonkova H*, et al.* Single Agent Daratumumab in Advanced Multiple Myeloma: Specific Healthcare Program in the Czech Republic. *Blood* 2017; **130**(suppl)**:** 5414.

53. Potluri R, Kanakamedala H, Chen C, Yasenchak CA, Ranjan S, Papademetriou E*, et al.* Duration of Treatment of Multiple Myeloma Regimens in Patients with Relapsed or Refractory Multiple Myeloma: Findings in US Clinical Practice Settings. *Blood* 2017; **130**(suppl)**:** 1844.

54. Varga G, Nagy Z, Kosztolányi S, Szomor A, Schneider T, Deák B*, et al.* Real World Data on the Efficacy and Safety of Ixazomib in Combination with Lenalidomide and Dexamethasone in Relapsed/Refractory Multiple Myeloma: Data Collected from the Hungarian Ixazomib Named Patient Program. *Blood* 2017; **130**(suppl)**:** 1865.

55. Terpos E, Maouche N, Minarik J, Katodritou E, Jenner MW, Plonkova H*, et al.* "Real World" Data on the Efficacy and Safety of Ixazomib in Combination with Lenalidomide and Dexamethasone in Relapsed/Refractory Multiple Myeloma: A Combined Study from the Greek, Czech and UK Databases. *Blood* 2017; **130**(suppl)**:** 3087.

56. Breckenridge ZM, Jimenez-Zepeda V, Bahlis NJ, Neri P, Sandhu I, Chu MP*, et al.* The Evolving Impact on Survival Outcomes in Multiple Myeloma with Greater Access to Novel Treatment Regimens: Experience in a Real-World Cohort. *Blood* 2017; **130**(suppl)**:** 1859.

57. Nooka A, Voorhees PM, Kumar SK, Mehra M, Lam A, Slavcev M*, et al.* Comparison of Overall Survival Associated with Lenalidomide+Dexamethasone and Bortezomib+Dexamethasone Among Relapsed/Refractory Multiple Myeloma Patients: A Matched Analysis of Real World and Clinical Trial Populations. *Blood* 2017; **130**(suppl)**:** 3094.

58. Rifkin RM, Medhekar R, Amirian ES, Wilson T, Boyd M, Mezzi K*, et al.* A Real-World Comparative Analysis of Carfilzomib and Other Systemic Multiple Myeloma Chemotherapies in the US Community Oncology Setting. *Blood* 2017; **130**(suppl)**:** 3434.

59. Nooka A, Voorhees PM, Kumar SK, Mehra M, Lam A, Slavcev M*, et al.* Evaluation of Efficacy Outcomes Among Relapsed/Refractory Multiple Myeloma Treated Patients in a Real-World Setting. *Blood* 2017; **130**(suppl)**:** 3093.

60. Ozsan GH, Sevindik OG, Sadri S, Hacioglu SK, Kaya SY, Geduk A*, et al.* Real-World Data in Relapsed Refractory Myeloma Patients Treated with Pomalidomide, a Multicenter Turkish Experience. *Blood* 2017; **130**(suppl)**:** 5430.

61. Sevindik OG, Pehlivan M, Tuglular TF, Solmaz SM, Dogu MH, Yalniz F*, et al.* Real-World Data in Relapsed Refractory Myeloma Patients Treated with Carfilzomib, a Multicenter Turkish Experience. *Blood* 2017; **130**(suppl)**:** 5405.

62. Chari A, Romanus D, Luptakova K, Raju A, Farrelly E, Blazer M*, et al.* Duration of Therapy (DOT) and Time to Next Therapy (TTNT) of Bortezomib, Carfilzomib and Ixazomib Combinations with Lenalidomide/Dexamethasone (VRd, KRd, IRd) in Patients (pts) with Relapsed/Refractory Multiple Myeloma (RRMM): Clinical Practice in the United States Vs Clinical Trial Experience. *Blood* 2017; **130**(suppl)**:** 1818.

63. Richardson PG, Sonneveld P, Schuster M, Irwin D, Stadtmauer E, Facon T*, et al.* Extended follow-up of a phase 3 trial in relapsed multiple myeloma: final time-to-event results of the APEX trial. *Blood* 2007 Nov 15; **110**(10)**:** 3557-3560.

64. Richardson PG, Sonneveld P, Schuster MW, Irwin D, Stadtmauer EA, Facon T*, et al.* Bortezomib or high-dose dexamethasone for relapsed multiple myeloma. *N Engl J Med* 2005 Jun 16; **352**(24)**:** 2487-2498.

65. Arnulf B, Pylypenko H, Grosicki S, Karamanesht I, Leleu X, van de Velde H*, et al.* Updated survival analysis of a randomized phase III study of subcutaneous versus intravenous bortezomib in patients with relapsed multiple myeloma. *Haematologica* 2012 Dec; **97**(12)**:** 1925-1928.

66. Moreau P, Pylypenko H, Grosicki S, Karamanesht I, Leleu X, Grishunina M*, et al.* Subcutaneous versus intravenous administration of bortezomib in patients with relapsed multiple myeloma: a randomised, phase 3, non-inferiority study. *Lancet Oncol* 2011 May; **12**(5)**:** 431-440.

67. Siegel DS, Oriol A, Rajnics P, Minarik J, Hungria V, Lee JH*, et al.* Updated Results from ASPIRE and ENDEAVOR, Randomized, Open-Label, Multicenter Phase 3 Studies of Carfilzomib in Patients (Pts) with Relapsed/Refractory Multiple Myeloma (RRMM). *Proceedings of the 2017 International Myeloma Workshop (IMW)* 2017; **16th IMW**(abstract PS-254)**:** e211-e212.

68. Stewart AK, Rajkumar SV, Dimopoulos MA, Masszi T, Spicka I, Oriol A*, et al.* Carfilzomib, lenalidomide, and dexamethasone for relapsed multiple myeloma. *N Engl J Med* 2015 Jan 08; **372**(2)**:** 142-152.

69. Dimopoulos MA, Moreau P, Palumbo A, Joshua D, Pour L, Hajek R*, et al.* Carfilzomib and dexamethasone versus bortezomib and dexamethasone for patients with relapsed or refractory multiple myeloma (ENDEAVOR): a randomised, phase 3, open-label, multicentre study. *Lancet Oncol* 2016 Jan; **17**(1)**:** 27-38.

70. Hajek R, Masszi T, Petrucci MT, Palumbo A, Rosinol L, Nagler A*, et al.* A randomized phase III study of carfilzomib vs low-dose corticosteroids with optional cyclophosphamide in relapsed and refractory multiple myeloma (FOCUS). *Leukemia* 2017 Jan; **31**(1)**:** 107-114.

71. Moreau P, Masszi T, Grzasko N, Bahlis NJ, Hansson M, Pour L*, et al.* Oral Ixazomib, Lenalidomide, and Dexamethasone for Multiple Myeloma. *N Engl J Med* 2016 Apr 28; **374**(17)**:** 1621-1634.

72. Dimopoulos MA, Lonial S, White D, Moreau P, Mateos MV, San Miguel J*, et al.* Phase 3 ELOQUENT-2 study: Extended 4-year follow-up of elotuzumab plus lenalidomide/dexamethasone vs lenalidomide/dexamethasone in relapsed/refractory multiple myeloma. *Haematologica* 2017; **102**(s2)**:** 167-168.

73. Lonial S, Dimopoulos M, Palumbo A, White D, Grosicki S, Spicka I*, et al.* Elotuzumab Therapy for Relapsed or Refractory Multiple Myeloma. *N Engl J Med* 2015 Aug 13; **373**(7)**:** 621-631.

74. Palumbo A, Chanan-Khan A, Weisel K, Nooka AK, Masszi T, Beksac M*, et al.* Daratumumab, Bortezomib, and Dexamethasone for Multiple Myeloma. *N Engl J Med* 2016 Aug 25; **375**(8)**:** 754-766.

75. Dimopoulos MA, Oriol A, Nahi H, San-Miguel J, Bahlis NJ, Usmani SZ*, et al.* Daratumumab, Lenalidomide, and Dexamethasone for Multiple Myeloma. *N Engl J Med* 2016 Oct 06; **375**(14)**:** 1319-1331.

76. San-Miguel JF, Hungria VT, Yoon SS, Beksac M, Dimopoulos MA, Elghandour A*, et al.* Panobinostat plus bortezomib and dexamethasone versus placebo plus bortezomib and dexamethasone in patients with relapsed or relapsed and refractory multiple myeloma: a multicentre, randomised, double-blind phase 3 trial. *Lancet Oncol* 2014 Oct; **15**(11)**:** 1195-1206.

77. San-Miguel JF, Hungria VT, Yoon SS, Beksac M, Dimopoulos MA, Elghandour A*, et al.* Overall survival of patients with relapsed multiple myeloma treated with panobinostat or placebo plus bortezomib and dexamethasone (the PANORAMA 1 trial): a randomised, placebo-controlled, phase 3 trial. *Lancet Haematol* 2016 Nov; **3**(11)**:** e506-e515.

78. Dimopoulos M, Spencer A, Attal M, Prince HM, Harousseau JL, Dmoszynska A*, et al.* Lenalidomide plus dexamethasone for relapsed or refractory multiple myeloma. *N Engl J Med* 2007 Nov 22; **357**(21)**:** 2123-2132.

79. Dimopoulos MA, Chen C, Spencer A, Niesvizky R, Attal M, Stadtmauer EA*, et al.* Long-term follow-up on overall survival from the MM-009 and MM-010 phase III trials of lenalidomide plus dexamethasone in patients with relapsed or refractory multiple myeloma. *Leukemia* 2009 Nov; **23**(11)**:** 2147-2152.

80. Weber DM, Chen C, Niesvizky R, Wang M, Belch A, Stadtmauer EA*, et al.* Lenalidomide plus dexamethasone for relapsed multiple myeloma in North America. *N Engl J Med* 2007 Nov 22; **357**(21)**:** 2133-2142.

81. San Miguel J, Weisel K, Moreau P, Lacy M, Song K, Delforge M*, et al.* Pomalidomide plus low-dose dexamethasone versus high-dose dexamethasone alone for patients with relapsed and refractory multiple myeloma (MM-003): a randomised, open-label, phase 3 trial. *Lancet Oncol* 2013 Oct; **14**(11)**:** 1055-1066.

82. Dimopoulos MA, Palumbo A, Corradini P, Cavo M, Delforge M, Di Raimondo F*, et al.* Safety and efficacy of pomalidomide plus low-dose dexamethasone in STRATUS (MM-010): a phase 3b study in refractory multiple myeloma. *Blood* 2016 Jul 28; **128**(4)**:** 497-503.

83. Moreau P, Joshua D, Chng WJ, Palumbo A, Goldschmidt H, Hajek R*, et al.* Impact of prior treatment on patients with relapsed multiple myeloma treated with carfilzomib and dexamethasone vs bortezomib and dexamethasone in the phase 3 ENDEAVOR study. *Leukemia* 2017 Jan; **31**(1)**:** 115-122.

84. de Waal EG, de Munck L, Hoogendoorn M, Woolthuis G, van der Velden A, Tromp Y*, et al.* Combination therapy with bortezomib, continuous low-dose cyclophosphamide and dexamethasone followed by one year of maintenance treatment for relapsed multiple myeloma patients. *Br J Haematol* 2015 Dec; **171**(5)**:** 720-725.

85. Kropff M, Bisping G, Schuck E, Liebisch P, Lang N, Hentrich M*, et al.* Bortezomib in combination with intermediate-dose dexamethasone and continuous low-dose oral cyclophosphamide for relapsed multiple myeloma. *Br J Haematol* 2007 Aug; **138**(3)**:** 330-337.

86. Dimopoulos MA, Stewart AK, Masszi T, Spicka I, Oriol A, Hajek R*, et al.* Carfilzomib-lenalidomide-dexamethasone vs lenalidomide-dexamethasone in relapsed multiple myeloma by previous treatment. *Blood Cancer J* 2017 Apr 21; **7**(4)**:** e554.

87. Garderet L, Iacobelli S, Moreau P, Dib M, Lafon I, Niederwieser D*, et al.* Superiority of the triple combination of bortezomib-thalidomide-dexamethasone over the dual combination of thalidomide-dexamethasone in patients with multiple myeloma progressing or relapsing after autologous transplantation: the MMVAR/IFM 2005-04 Randomized Phase III Trial from the Chronic Leukemia Working Party of the European Group for Blood and Marrow Transplantation. *J Clin Oncol* 2012 Jul 10; **30**(20)**:** 2475-2482.
